# Can cardiac MRI be the 'crystal ball' for risk stratification in dilated cardiomyopathy? The impact of an LV mid-myocardial stripe on LVAD and transplantation risk

**DOI:** 10.1186/1532-429X-13-S1-O102

**Published:** 2011-02-02

**Authors:** Jose Venero, Srinivas Murali, Mark Doyle, Vikas K Rathi, Saundra B Grant, June A Yamrozik, Ronald B Williams, Diane Vido, Geetha Rayarao, Raymond Benza, George Sokos, David Dean, Robert WW Biederman

**Affiliations:** 1 Allegheny General Hospital, Pittsburgh, PA, USA; 2 Bon Secours Heart and Vascular Institute, Richmond, VA, USA

## Introduction

Patients with newly diagnosed dilated cardiomyopathy and advanced heart failure have a very high morbidity and mortality with an unpredictable clinical course. We investigated the role of CMR via LGE in this cohort of patients.

## Purpose

**Hypothesis **Utilizing cardiovascular MRI (CMR), via the late gadolinium enhancement (LGE), we assessed the prognostic value in primary dilated cardiomyopathy(DCM).

## Methods

Over 39 consecutive months, 51 cardiomyopathy patients were referred for standard 3D CMR(1·5T,GE) to interrogate the LV pattern, distribution and extent of DHE (MultiHance, Princeton, NJ). Inclusion criteria were met in 21 patients. DCM were categorized into: 1) +midwall Stripe 2)-Stripe groups. Major adverse clinical events (MACE), mortality, need for LV assist device (LVAD), and urgent cardiac transplantation (Tx), were evaluated over the ensuing 6 and 12 months.

## Results

All patients were alive at 12 months while 11/21(52%) demonstrated a +Stripe. There were no differences between groups for demographics, baseline LVEF, LV end-diastolic diameter, NYHA class or hemodynamics. The group with +Stripe categorization strongly predicted the need for LVAD and/or urgent Tx surgery over the ensuing 6 (or 12) months (p<0.005). Specifically, 7/11(74%) +Stripe patients required urgent Tx by 6 months, while no patients in the −Stripe group ever experienced the need for LVAD/urgent Tx (Kaplan-Meier: X^2^= 9, p<0.005). Similarly, +Stripe predicted MACE by Cox regression modeling (p<0.05). No patient in the −Stripe group had a MACE at 6 months (one rehospitalization at 12 months).

## Conclusions

The presence of +Stripe on CMR is predictive of LVAD and Tx need over the ensuing 6 months and remains robust out to 12 months in DCM patients. Incorporating CMR imaging into routine clinical practice may help to identify early those high risk DCM patients; conservatively manage low-risk pts while expectantly manage high-risk patients.

